# Structural Modeling and Theoretical Study of Low-Dimensional Materials

**DOI:** 10.3390/nano16211423

**Published:** 2026-11-04

**Authors:** Xiao-Chun Wang, Xilian Jin

**Affiliations:** 1 School of Physics Science and Information Technology, Liaocheng University, Liaocheng 252000, China; 2 State Key Laboratory of High Pressure and Superhard Materials, College of Physics, Jilin University, Changchun 130012, China

## 1. Introduction

Since the experimental isolation of single-layer graphene in 2004 [1], one-dimensional (1D) and two-dimensional (2D) materials have become one of the most active frontiers in condensed-matter physics and materials science. The family of 2D materials now extends well beyond graphene to transition-metal dichalcogenides (TMDCs), phosphorene, hexagonal boron nitride (h-BN), Janus membrane, and pentagonal carbon allotropes, while carbon nanotube 1D materials and their derivatives, including helical single-walled carbon nanotubes (SWCNTs) and carbon nanorings (CNRs), continuously enrich the nanoscale material library. Owing to their exceptional electronic, optical, mechanical, and thermal properties, these materials show great promise for next-generation electronics, optoelectronics, flexible and wearable devices, energy conversion, and impact-protection systems [2,3,4].

To fully exploit these materials, precise prediction and modulation of their physical properties are essential. Computational approaches, including first-principle calculations, continuum mechanics, and molecular dynamics (MD) simulations, have become indispensable tools that reveal the microscopic mechanisms underlying nanoscale behavior, ranging from nonlocal and interlayer interactions to spin-orbit coupling and multiphonon scattering, and guide the rational design of experiments. In parallel, surface-functionalized nanomaterial composites are continuously advancing engineering applications, such as insulating materials, for power equipment. Rich degrees of freedom, including strain, twist angle, stacking order, electric field, doping, and size, provide versatile knobs for property engineering.

This Special Issue brings together nine articles dedicated to 1D and 2D nanomaterials, covering their electronic, optical, mechanical, thermal, and energy-related properties. The collection spans exact analytical modeling of helical SWCNT beams, a novel finite-element approach to the buckling of carbon nanorings, first-principle design of piezoelectric and thermoelectric 2D materials, continuum-model studies of nonlinear optical responses, coarse-grained simulations of ballistic protection, and experimental co-fluorinated graphene composites for high-voltage insulation. Our Special Issue is expected to promote and accelerate ongoing research efforts on 1D and 2D nanomaterials, and will be of interest to the general readers of *Nanomaterials*.

## 2. An Overview of Published Articles

Dalgıç et al. [5] presented an exact analytical investigation of the static response of helical single-walled carbon nanotube beams based on Eringen’s nonlocal Euler–Bernoulli beam theory, which captures nanoscale effects arising from interatomic interactions. To the best of the authors’ knowledge, this represents the first closed-form formulation for such complex nanostructures within this theoretical framework. Considering cantilevered and clamped–clamped boundary conditions under concentrated force and moment loadings, and expressing displacements and rotations in the Frenet frame, they carried out parametric studies of the winding angle, aspect ratio, and nonlocal parameter. The results show that nonlocal elasticity theory consistently predicts larger displacements and rotations than the classical local theory, revealing its importance for accurate modeling of nanoscale structures; the proposed analytical framework serves as a benchmark reference for the modeling and design of nano-springs, actuators, and flexible nanodevices.

Liu et al. [6] designed and predicted, using first-principles calculations, stable 2D X-penta-graphene (X = Si or Ge) semiconductors with excellent in-plane and out-of-plane piezoelectricity and extremely high in-plane hole mobility. Among them, Ge-PG exhibits superior performance, with an in-plane piezoelectric coefficient d_11_ = 8.43 pm/V, an out-of-plane coefficient d_33_ = −3.63 pm/V, and an in-plane hole mobility μ_h_ = 57.33 × 10^3^ cm^2^ V^−1^ s^−1^. The substitution of Si and Ge atoms gradually weakens the mechanical strength of the structure, causing the negative Poisson’s ratio of penta-graphene to approach zero and then invert to a positive value, while the band gaps of Si-PG (0.78 eV) and Ge-PG (0.89 eV) are significantly reduced compared with that of penta-graphene (2.20 eV). Importantly, the physical mechanism of the out-of-plane piezoelectricity is revealed: the super-dipole-moment effect proposed in previous work is confirmed to exist in penta-graphene and X-PG, with the out-of-plane piezoelectric stress coefficient e_33_ increasing with the super-dipole-moment and the induced polarization direction consistent with it. X-PG is predicted to hold prominent potential for electromechanical coupling systems, wearable ultra-thin devices, and high-speed electronic transmission devices.

Wang et al. [7] introduced the Chebyshev–Ritz method, a novel finite-element approach, to investigate the buckling behaviors of carbon nanorings as an alternative to molecular dynamic simulations. With carefully selected mechanical parameters, the critical deformation of a carbon nanoring with 50 repeated units determined by the Chebyshev–Ritz method aligns with an MD simulation presenting a buckling number of 14. Moreover, using a constant mechanical parameter for 50 repeated units, a structural transition at varying toric sizes is observed, leading to the stabilization of buckling numbers 13, 14, and 15, which has also been corroborated by MD simulations. Their approach offers a reliable and accurate means of examining the structural properties of large-scale nanomaterials and paves the way for further exploration in nanoscale mechanics.

Dong et al. [8] employed a combined treatment using plasma and a fluorine-containing coupling agent to co-fluorinate graphene nanosheets (GNSs), obtaining fluorinated graphene nanosheets (DFGNSs), and prepared GNSs/DFGNS-modified epoxy resin (EP) composites with different loadings to enhance their surface insulation properties. Tests on surface flashover voltage, surface charge distribution, and surface resistivity demonstrated that both GNSs and DFGNSs significantly improve the insulation properties of EP materials, with the optimal improvement achieved at a DFGNS content of 0.2 wt%, where the flashover voltage increased by 16.23%. The work provides an effective strategy for developing high-performance insulating composites to meet the increasing performance demands of high- and ultra-high-voltage power transmission and transformation equipment.

Chen et al. [9] theoretically investigated the comprehensive modulation of interlayer twisting and external electric fields on the two-photon absorption (TPA) of a twisted graphene/hexagonal boron nitride (tG/hBN) heterojunction with small twist angles (2° < θ < 10°), starting from an effective continuum model. It was found that the TPA of tG/hBN extended from the infrared band of twisted bilayer graphene to the visible band due to the increase in the energy band gap caused by twisting and the potential energy of the boron nitride atomic layer. Furthermore, the TPA coefficient is enhanced several times by an external vertical electric field, which increases the density of states and thus the transition probability for two-photon absorption. The findings provide theoretical guidance for nonlinear nanophotonic and optoelectronic applications of twisted van der Waals heterostructures.

Yu et al. [10] investigated strain-, twist-, and electric-field-tuned optical absorption in bilayer MoS_2_, emphasizing spin–orbit coupling (SOC). A continuum model reveals competing mechanisms: geometric perturbations (strain/twist) and Stark effects govern one-/two-photon absorption, with critical thresholds (~9% strain and ~2.13° twist) switching from spin-independent to spin-polarized regimes. Strain gradients and twist enhance nonlinear responses through symmetry-breaking effects, while electric fields dynamically modulate absorption via band alignment tuning. By linking parameter configurations to absorption characteristics, this work provides a framework for designing tunable spin-resolved optoelectronic devices and advancing light-matter control in 2D materials.

Ren et al. [11] constructed four types of 2D hexagonal BP monolayer (h-BP)/borophosphene van der Waals heterostructures with different stacking orders, namely B-B stacking, P-P stacking, moiré-I, and moiré-II, and explored their structural stability and electronic and optical properties using first-principle calculations. All heterostructures maintain good structural stability with minimal lattice mismatch and exhibit semiconducting characteristics, with band gaps highly dependent on the interlayer stacking order. Owing to the regular atomic arrangement and enhanced interlayer dipole interactions, the B-B stacking bilayer opens a relatively large band gap of 0.157 eV, while the moiré-II bilayer exhibits a very small band gap of 0.045 eV because of its irregular atomic arrangements. The optical absorption spectra of the B-B and P-P stacking bilayers were further discussed, suggesting that h-BP/borophosphene heterostructures possess desirable optical properties that broaden the potential applications of the constituent monolayers.

Lan et al. [12] systematically investigated the thermal conductivity and thermoelectric properties of penta-XP_2_ (X = Pd, Pt) monolayers through first-principle calculations, incorporating four-phonon (4ph) scattering and electron-phonon interaction (EPI) effects. The 4ph scattering, particularly Umklapp and redistribution processes, markedly suppresses the lattice thermal conductivity by generating substantial thermal resistance and disrupting phonon population distributions: at 300 K, the lattice thermal conductivity is reduced to 0.87 W/mK (80% reduction) for penta-PdP_2_ and 1.64 W/mK (79% reduction) for penta-PtP_2_ compared with three-phonon-only scattering. Combined with EPI-optimized electronic transport, the thermoelectric figures of merit (ZT) increase from 0.21 to 0.86 for penta-PdP_2_ and from 0.11 to 0.34 for penta-PtP_2_, alongside a broadened optimal carrier concentration range. These findings highlight momentum-conserving 4ph scattering as a key mechanism for phonon transport modulation and thermoelectric efficiency improvement, providing theoretical guidance for designing high-performance nanoscale thermal management and energy conversion devices.

Liu et al. [13] investigated the coupling mechanism between the interlayer twist angle and projectile size on the ballistic performance of bilayer phosphorene membranes using coarse-grained molecular dynamic simulations, systematically exploring twist angles in the range of 0–90° and projectile radii in the range of 2–10 nm for membranes with a radius of 48 nm. The effect of the twist angle was found to become significant only beyond a critical projectile size of ~8 nm; below this threshold, deformation remains local and twist-independent, whereas for larger projectiles, the twist angle drastically alters wave propagation and failure modes. Specifically, a 90° twist induces severe wave reflection and interference, leading to a dramatic force amplification of up to 82% and a 28% reduction in the ballistic limit velocity, making it the most susceptible configuration. These results underline the critical role of the twist–boundary–wave interaction in governing impact resistance and provide practical insights for the design of phosphorene-based nano-armor systems tailored to specific impact conditions.

## 3. Conclusions

The articles collected in this Special Issue address key challenges in the electronic, optical, mechanical, thermal, and energy-related properties of one- and two-dimensional nanomaterials. The majority of the contributions employ computational approaches, including exact continuum analytical solutions, first-principle calculations, and (coarse-grained) molecular dynamic simulations, demonstrating their power in predicting material properties and revealing the underlying microscopic mechanisms, from nonlocal interactions and the super-dipole-moment effect to stacking-dependent interlayer dipoles, four-phonon scattering, and twist–boundary–wave coupling. One experimental study further bridges functionalized nanomaterial composites with engineering applications in high-voltage insulation.

These advances may accelerate the design of next-generation nanoscale systems, including flexible and wearable electronics, spin-resolved optoelectronics, nano-springs and actuators, thermal management and thermoelectric devices, impact-protective membranes, and insulating composites for power equipment. The Guest Editors anticipate that this Special Issue will foster and accelerate current research activities concerning low-dimensional nanomaterials, and that it will attract the attention of *Nanomaterials*’ wider audience.

## References

[1] Novoselov K.S., Geim A.K., Morozov S.V., Jiang D., Zhang Y., Dubonos S.V., Grigorieva I.V., Firsov A.A. (2004). Electric Field Effect in Atomically Thin Carbon Films. Science.

[2] Tang D.-S., Luo Y.-Q., Zhu D.-Y., Wang J.-H., Shao X.-T., Cui S.-X., Wang X.-C. (2025). Enhanced piezoelectricity in TiSXY monolayers based on electronegative polar moments effect. Appl. Phys. Lett..

[3] He Q.-W., Wang J.-H., Zhu D.-Y., Tang D.-S., Lv Z., Guo F., Wang X.-C. (2024). Strong Vertical Piezoelectricity and Broad-pH-Value Photocatalyst in Ferroelastic Y2Se2BrF Monolayer. Nano Lett..

[4] Liu A., Zhang X., Liu Z., Li Y., Peng X., Li X., Qin Y., Hu C., Qiu Y., Jiang H. (2024). The Roadmap of 2D Materials and Devices Toward Chips. Nano-Micro Lett..

[5] Dalgıç A.M., Tüfekci M., Pir İ., Tüfekci E. (2025). Exact Analytical Solutions for Static Response of Helical Single-Walled Carbon Nanotubes Using Nonlocal Euler–Bernoulli Beam Theory. Nanomaterials.

[6] Liu S., Shang X., Liu X., Wang X., Liu F., Zhang J. (2024). Excellent Hole Mobility and Out–of–Plane Piezoelectricity in X–Penta–Graphene (X = Si or Ge) with Poisson’s Ratio Inversion. Nanomaterials.

[7] Wang X., Kuang G., Tian H., Shao Z., Dong N., Lin T., Huang L. (2024). Investigation of Buckling Behaviors in Carbon Nanorings Using the Chebyshev–Ritz Method. Nanomaterials.

[8] Dong M., Yang Z., Xia G., Zhang J., Zhan Z., Xin W., Wang Q., Xu B., Zhang Y., Xie J. (2024). Enhance the Surface Insulation Properties of EP Materials via Plasma and Fluorine-Containing Coupling Agent Co-Fluorinated Graphene. Nanomaterials.

[9] Chen M., Chen Y., Yang G., Wang Q., Feng X. (2025). Two-Photon Absorption in Twisted Graphene/Hexagonal Boron Nitride Heterojunction Tuned by Vertical Electric Field. Nanomaterials.

[10] Yu L., Chen Y., Zhang W., Yang P., Feng X. (2025). Spin-Orbit-Coupling-Governed Optical Absorption in Bilayer MoS2 via Strain, Twist, and Electric Field Engineering. Nanomaterials.

[11] Ren K., Zhang Q., Zhang S., Zhang Y. (2025). Stacking Order-Dependent Electronic and Optical Properties of h-BP/Borophosphene Van Der Waals Heterostructures. Nanomaterials.

[12] Lan Y., Zhang Y., Zhang H., Wang P., Wang N., Yan Y., Zha X., Ding C., Li Y., Li C. (2025). Impact of Four-Phonon Scattering on Thermal Transport and Thermoelectric Performance of Penta-XP2 (X = Pd, Pt) Monolayers. Nanomaterials.

[13] Liu N., Huang K., Yang X., Xu D., Wang L. (2025). Mechanism of Coupling Twist Angle and Projectile Radius on Ballistic Impact Performance of Bilayer Phosphorene Membranes. Nanomaterials.

